# Inhibition of let-7b-5p contributes to an anti-tumorigenic macrophage phenotype through the SOCS1/STAT pathway in prostate cancer

**DOI:** 10.1186/s12935-020-01563-7

**Published:** 2020-09-29

**Authors:** Jiping Rong, Lu Xu, Yinying Hu, Fan Liu, Yanrong Yu, Hongyan Guo, Xudong Ni, Yanqin Huang, Lin Zhao, Zhigang Wang

**Affiliations:** 1 Jiangxi Academy of Medical Sciences, and Jiangxi Provincial Key Laboratory of Immunotherapy, Nanchang, China; 2 Medical College of Nanchang University, Nanchang, China

**Keywords:** Let-7b-5p, SOCS1/STAT pathway, Macrophage polarization, Prostate cancer

## Abstract

**Background:**

Dysfunction of microRNAs (miRNAs) is a major cause of aberrant expression of inflammatory cytokines and contributes to macrophage polarization. Proinflammatory M1 macrophages promote T helper (Th) 1 responses and show tumoricidal activity, whereas M2 macrophages display regulatory functions in tissue repair and remodeling and promote Th2 immune responses. Previous studies have shown that miRNA let-7 is associated with cellular differentiation and that the expression of let-7b-5p is significantly augmented in M2 macrophages. However, the mechanism by which let-7b-5p regulates macrophage differentiation in prostate cancer (PCa) remains largely unknown.

**Methods:**

Human macrophages were induced by blood monocytes from healthy male donors, and M1 macrophages were polarized by stimulating them overnight with 100 ng/ml of lipopolysaccharides and 100 ng/ml of IFN-γ. Conditioned medium from PC-3 cells was used to induce prostatic macrophages (M-CMs) in vitro, and we then transfected let-7b-5p mimics or inhibitors into M1 and M-CMs for 72 h. The expression of cluster of differentiation 206 (CD206) in each group was detected with the High-Throughput Connotation of Imaging System. We used quantitative real-time polymerase chain reaction (qRT-PCR) to examine the expression of the inflammatory cytokines IL-10, IL-12, IL-13, TNF-alpha, and let-7b in macrophages. SOCS1 protein levels were evaluated by ELISA, and the phosphorylation difference in STAT family member proteins was analyzed using CST signal-pathway chip. Phagocytosis by macrophages and the effect of macrophages on the proliferation of prostate cancer PC-3 cells were evaluated with phagocytosis assay or the Cell Counting Kit-8 (CCK-8) and colony formation assay. The relationship between SOCS1 and let-7b-5p was confirmed with a dual-luciferase reporter.

**Results:**

The expression of cluster of differentiation 206 (CD206, a M2-like macrophage surface molecule) was significantly increased in M1 macrophages treated with let-7b-5p mimics, while CD206 expression was decreased in M-CMs treated with let-7b-5p inhibitors. Overexpression or knockdown of let-7b-5p significantly affected the expression of inflammatory factors in macrophages—including interleukin 10 (IL-10), IL-12, IL-13, and tumor necrosis factor alpha. Let-7b-5p downregulated the expression of suppressor of cytokine signaling 1 (SOCS1) and increased the phosphorylation of signal transducer and activator of transcription 1 (STAT1), STAT3, and STAT5a proteins in M-CMs and M1 macrophages with let-7b-5p mimics relative to the other groups. In addition, with the elevated expression of let-7b-5p, the phagocytosis by macrophages showed a commensurate and significant decrease. As a result, M-CMs treated with let-7b-5p inhibitors reduced the proliferation of PC-3 PCa cells.

**Conclusions:**

Collectively, these data indicated that let-7b-5p may regulate M2 polarization through the SOCS1/STAT pathway and that reversal of M2 differentiation by let-7b-5p inhibitors enhanced macrophage phagocytosis, ultimately inhibiting the proliferation of PCa cells.

## Background

Prostate cancer (PCa) is the second-most common cause of cancer-related deaths in men in the western world [1, 2]. Recent advances have revealed that the tumor microenvironment (TME) is an important determinant of tumor behavior and that cancer cells are confronted with various types of stromal and immune cells across all stages of disease progression. Increasing evidence has indicated that the “cross-talk” between tumors and stromal cells can modify cellular compartments, leading to the co-evolution of tumor cells and the TME. Macrophages play a prominent and active role in the TME, infiltrating tumors and actively contributing to the initiation and progression of cancer. There are recent reports of tumors being infiltrated by macrophages that possess proangiogenic activity, and that are generally associated with high vascular density [3]. Increased infiltration of tumor-associated macrophages (TAMs) has also been associated with worsening pathologic characteristics and a poor prognosis in breast, colon, and bladder cancer [4, 5].

Macrophages are classified into classically activated M1-like or alternatively activated M2-like macrophages based on their functional status as induced by the microenvironment. The majority of evidence indicates that TAMs within the primary TME are considered to be similar to M2 macrophages and play a tumor-promoting role that inhibits inflammation and promotes tumor invasion and metastasis [6]. Moreover, macrophages account for most of the infiltrating immune cells in the TME, comprising up to 50% of the tumor mass [7].

A recent study revealed that the distribution and function of TAMs differ considerably in different microregions of the neoplastic tissue, and macrophages have a mixed phenotype—expressing both M1- and M2-like markers [8]. Different signals from specific components of the TME seem to influence the activation of TAMs, and many inflammatory-signaling pathways reportedly play a role in macrophage polarization; however, how these signals are terminated or decay is unknown. Suppressor of cytokine signaling (SOCS) proteins are negative feedback regulators of the Janus kinase/signal transducer and activator of transcription (JAK/STAT) or receptor tyrosine kinase pathways [9] that mediate cytokine-induced immunologic responses, and it has been demonstrated that abnormal expression of SOCS is involved in the occurrence and progression of human cancers. For example, SOCS1 exerts growth-inhibiting functions through the downregulation of cyclins and cyclin-dependent kinases in PCa; SOCS proteins also act as regulators of innate and acquired immunity, negatively regulating the state of macrophages and dendritic cells (DCs) [10].

MicroRNAs (miRNAs) are master epigenetic regulators that are involved in the initiation and progression of various tumors [11, 12]. In immune cells that infiltrate tumors, miRNAs can exert effects on cellular function and phenotype, and can then enhance or suppress anti-tumor immunity by mediating some immune-regulating molecules [13]. The let-7 miRNA family regulates developmental timing and cellular proliferation, mediates immune responses, and adjusts inflammation [14, 15]. Members of this family have been identified as regulators of immune escape by directly or indirectly modulating the expression of immune-regulating molecules—especially important cytokines such as interleukin 6 (IL-6), IL-10, and tumor necrosis factor alpha (TNF-α) [16]. However, the relationship between let-7 and SOCS/STAT in macrophage differentiation remains unclear.

Our previous results indicated that conditioned media from PC-3 PCa cells induced macrophages to differentiate into M2 macrophages, a process that is significantly associated with the high expression of let-7 [17]. Therefore, we herein focused on let-7b-5p targeting of SOCS1/STAT in macrophages and its role in the progression of PCa. Our results showed that let-7b-5p has the capacity to promote macrophage differentiation to M2 by regulating the SOCS1/STAT pathway, and that reversal of M-CMs by let-7b-5p inhibitors led to significantly increased phagocytosis and suppressed the proliferation of PCa cells.

## Results

### Let-7b-5p promotes expression of cluster of differentiation 206 in macrophages

**Fig. 1:**
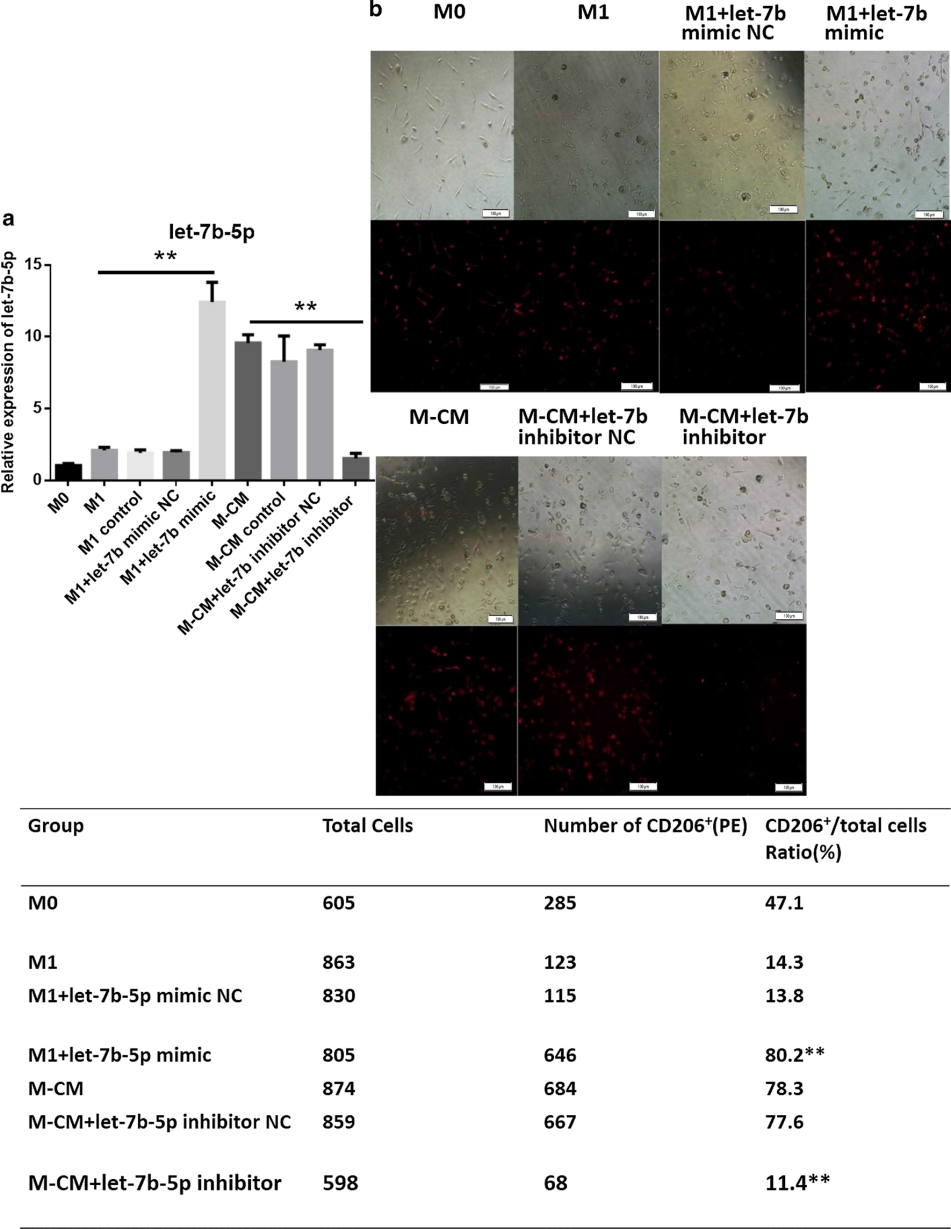
Let-7b-5p enhances CD206 expression in macrophages. **a** Let-7b-5p expression in different macrophages. Human monocytes were isolated from normal donor blood using anti-CD14 magnetic beads, and M0, M1, and M-CMs were differentiated as described in “Materials and methods”. M-CMs were transfected with let-7b-5p inhibitors or NC, or remained untreated for 72 h; M1 macrophages were transfected with let-7b-5p mimics. Relative expression of let-7b-5p was analyzed with qPCR. The expression of miRNA was normalized to U6. Data represent the mean ± standard deviation (SD) of 3 independent experiments. *P < 0.05; **P < 0.01. **b** CD206 expression in macrophages. After M1 and M-CMs were transfected with let-7b-5p mimics or inhibitors, respectively, CD206 expression of macrophage subtypes was detected with anti-CD-206 PE (red) by the High Throughput Connotation of Imaging System (original magnification, 100×)

Cluster of differentiation 206 (CD206) is a mannose receptor on the surface of macrophages and a specific marker of M2 macrophages. To evaluate the relationship between CD206 and let-7b-5p in macrophages, we first obtained M0, M1, and M-CMs from human blood monocytes (isolated from healthy male donors) as described in “Materials and methods” and then transfected let-7b-5p mimics or inhibitors into M1 macrophages or M-CMs, respectively. Subsequently, we investigated CD206 expression by the macrophages using the High-Throughput Connotation of Imaging System. Our results showed that these let-7b-5p mimics or inhibitors effectively regulated let-7b levels (Fig. 1a): the expression of CD206 in M-CMs (11.4%) treated with let-7b-5p inhibitors was almost identical to that in M1 macrophages (14.3%), whereas CD206 levels in M1 treated with let-7b-5p mimics (80.2%) were similar to those of M-CMs (78.3%) (Fig. 1b). These data indicated that let-7b-5p promoted CD206 expression in macrophages.

### Let-7b-5p regulates the expression of inflammatory cytokines in macrophages

**Fig. 2:**
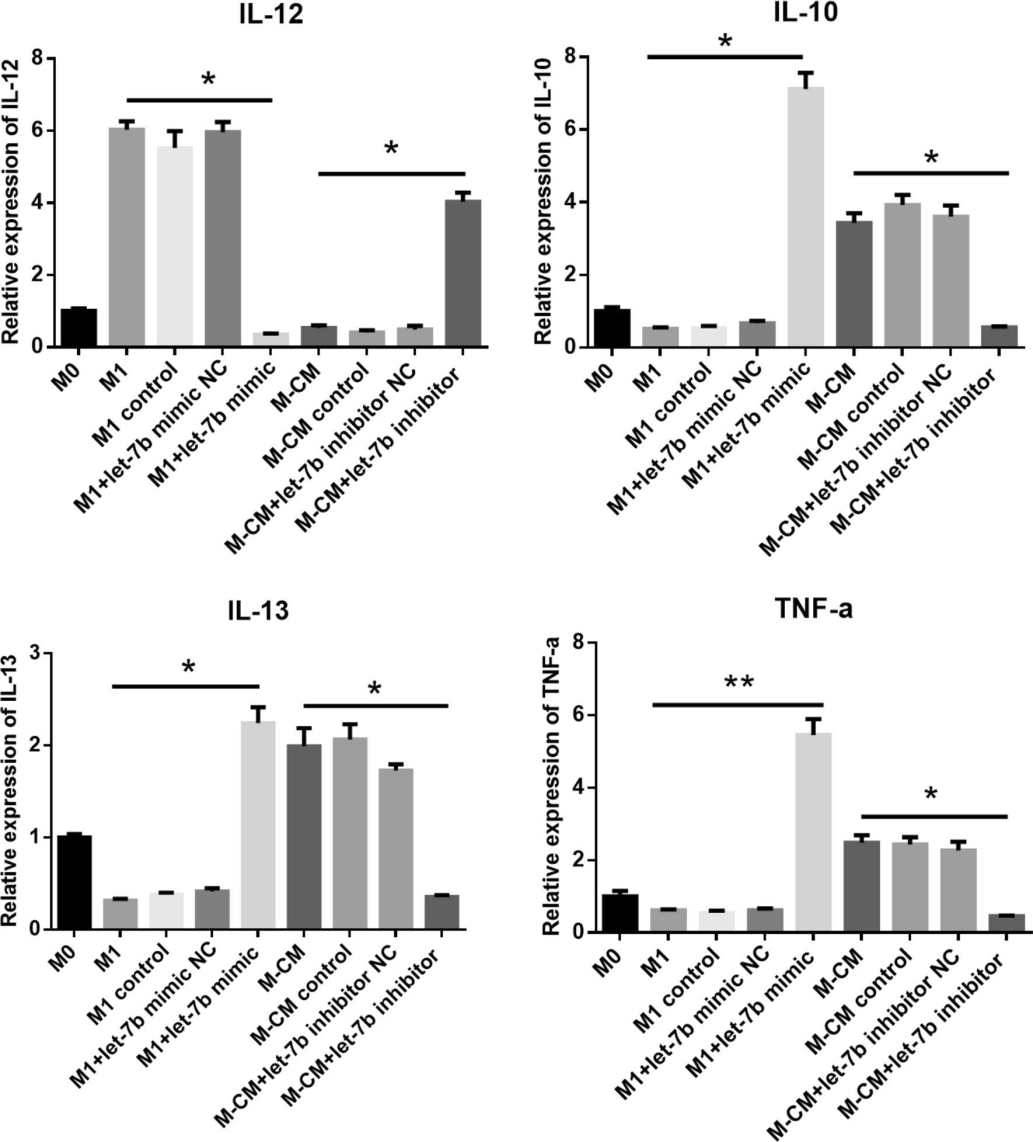
Effect of let-7b-5p on the expression of inflammatory cytokines in macrophages. M0, M1, and M-CMs were differentiated as described in “Materials and methods”. M-CMs were transfected with let-7b-5p inhibitors or NC, or remained untreated for 72 h; M1 macrophages were transfected with let-7b-5p mimics. Expression of IL-12, IL-13, IL-10, and TNF-α was determined by qPCR. Expression of mRNA was normalized to GAPDH. Data represent the mean ± SD of 3 separate experiments. *P < 0.05; **P < 0.01

To further investigate the role of let-7b-5p in the secretion of macrophage inflammatory cytokines, let-7b mimics or inhibitors were transfected into M1 and M-CMs, respectively. After 72 h, we analyzed the expression of inflammatory cytokines, including IL-12, IL-13, IL-10, and TNF-α. Our results revealed that after M-CMs were treated with let-7b-5p inhibitors, the expression of TNF-α, IL-10, and IL-13 was significantly decreased (**P < 0.01) similar to that for M1, whereas IL-12 was significantly upregulated. In addition, in the presence of let-7 mimics the levels of these cytokines in M1 displayed diametric results (Fig. 2). These data indicated that let-7b-5p modulated the expression of IL-12, IL-13, IL-10, and TNF-α in macrophage polarization.

### Let-7b-5p directly targets the SOCS1 3′-untranslated region

**Fig. 3:**
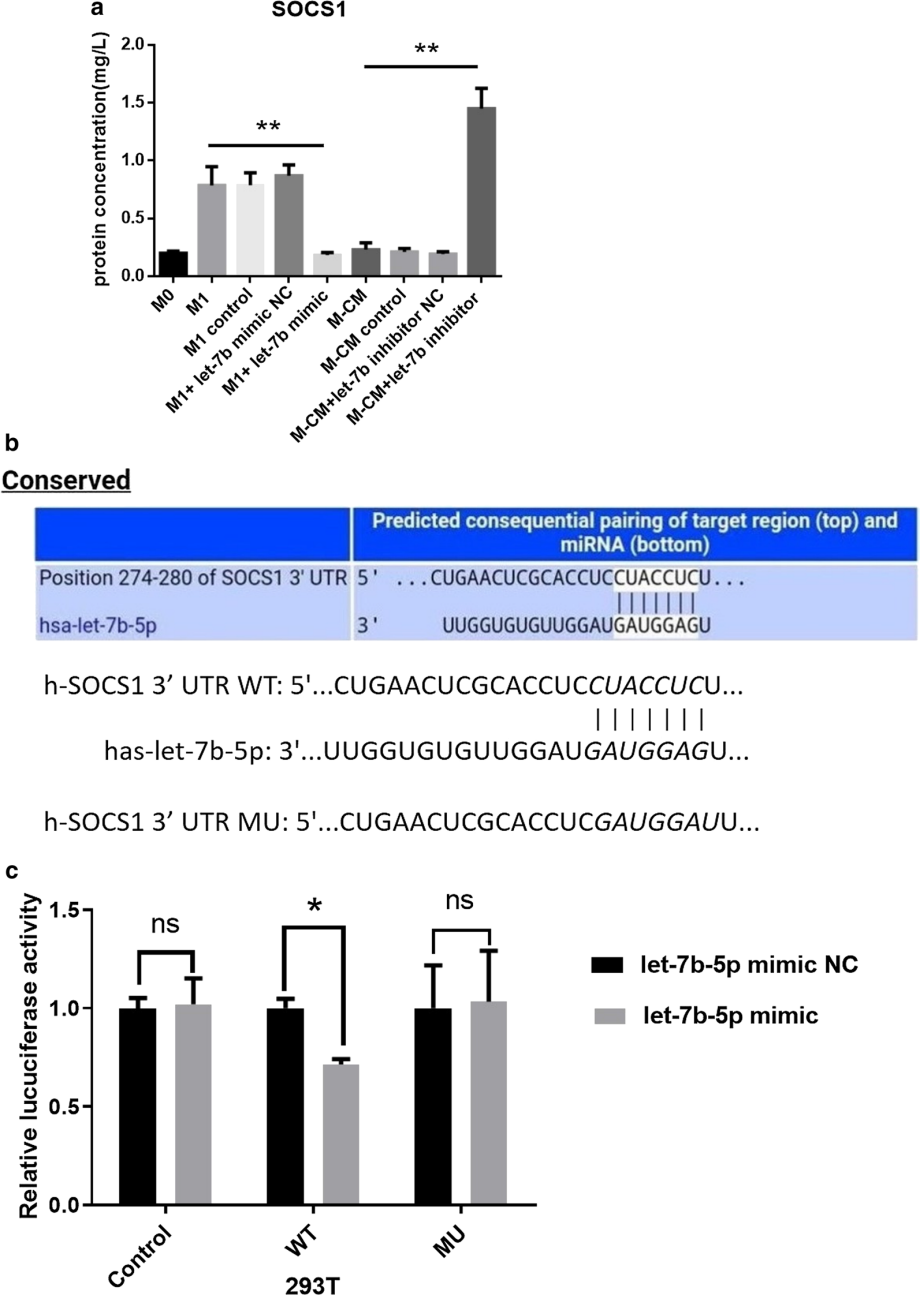
SOCS1 is a direct target gene of let-7b-5p in vitro. **a** Protein expression of SOCS1 in macrophages of each group. Expression of SOCS1 protein was confirmed by ELISA after M1 or M-CMs were transfected with let-7b-5p mimics or inhibitors, respectively, for 72 h, compared with the respective controls. **b** Illustration of the sequence match between let-7b-5p and SOCS1 3′-UTR as determined using TargetScan. **c** Relative luciferase activity was assayed and calculated by the ratio of firefly-*Renilla* luciferase activity following transfection with let-7b-5p mimic compared with transfection with NC in 293 T. The data are shown as the mean ± SD of 3 separate experiments. *P < 0.05; **P < 0.01

According to the predicted results with TargetScan, SOCS1 was considered to be the target of let-7b-5p. To demonstrate the relationship between let-7b-5p and SOCS1, we first detected SOCS1 expression in macrophages. Enzyme-linked immunoassay (ELISA) results showed decreased SOCS1 expression in M1 treated with let-7b-5p mimics, whereas the reverse occurred in M-CMs treated with let-7b-5p-inhibitors (Fig. 3a; P < 0.05). Next, we used a dual-luciferase reporter assay to evaluate whether SOCS1 is a direct target gene of let-7b-5p. Our data demonstrated that let-7b-5p markedly inhibited the activity of firefly luciferase in the wild-type (WT) group compared with the miR-negative control (NC), while this inhibitory effect was abolished among cells in the mutant (MUT) group (Fig. 3b, c; P < 0.05). These data suggested that let-7b-5p directly targets and regulates SOCS1 expression in macrophages.

### Let-7b-5p activates the STAT-signaling pathway

**Fig. 4:**
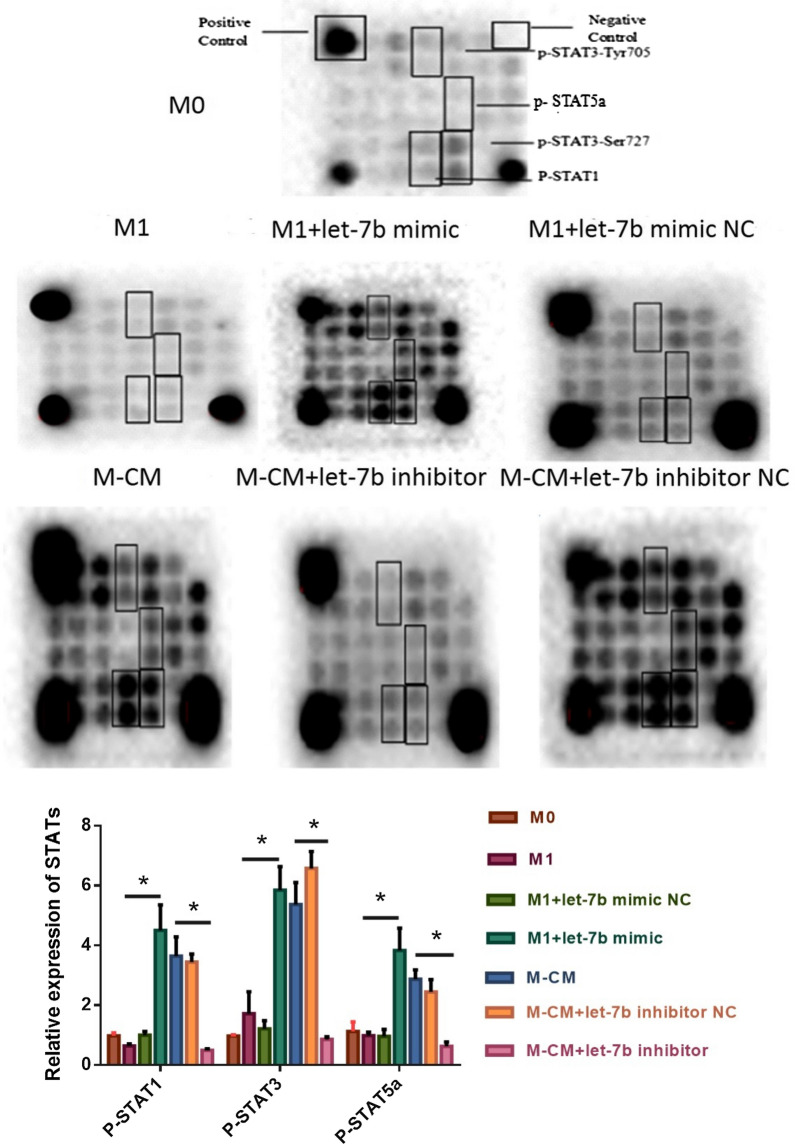
Overexpression or knockdown of let-7b-5p influences the activity of the STAT-signaling pathway. Macrophage cells of each group were harvested and lysed, and the supernatant was collected. After the protein concentration was determined using the Bio-Rad protein assay, 40 µg of cell lysates from each group was used to analyze the expression of the signaling proteins of macrophages using the CST signal-pathway chip according to the manufacturer’s instructions. These data were visualized with the Bio-Rad imager

To explore the mechanism underlying let-7b-5p regulation of macrophage polarization, we further analyzed the effects of let-7b-5p on the STAT-signaling pathway. After the transfection of M1 and M-CMs with let-7b-5p mimics or inhibitors, respectively, the CST chip was used to observe the changes in some signaling molecules. Our results showed that let-7b-5p promoted the phosphorylation of STAT1, STAT3, and STAT5A proteins in macrophages (Fig. 4). In M1 transfected with let-7b-5p mimics, the expression of phosphorylated STAT1 (p-STAT1), p-STAT5a, and p-STAT3 was significantly increased (**P < 0.01)—similar to the M-CM group. In contrast, the expression of p-STAT1, p-STAT3, and p-STAT5a was significantly decreased in M-CMs transfected with let-7b-5p inhibitors (**P < 0.01)—similar to that in the M1 group. These results indicated that let-7b-5p activates the STAT-signaling pathway.

### Let-7b-5p reduces phagocytosis by macrophages

**Fig. 5:**
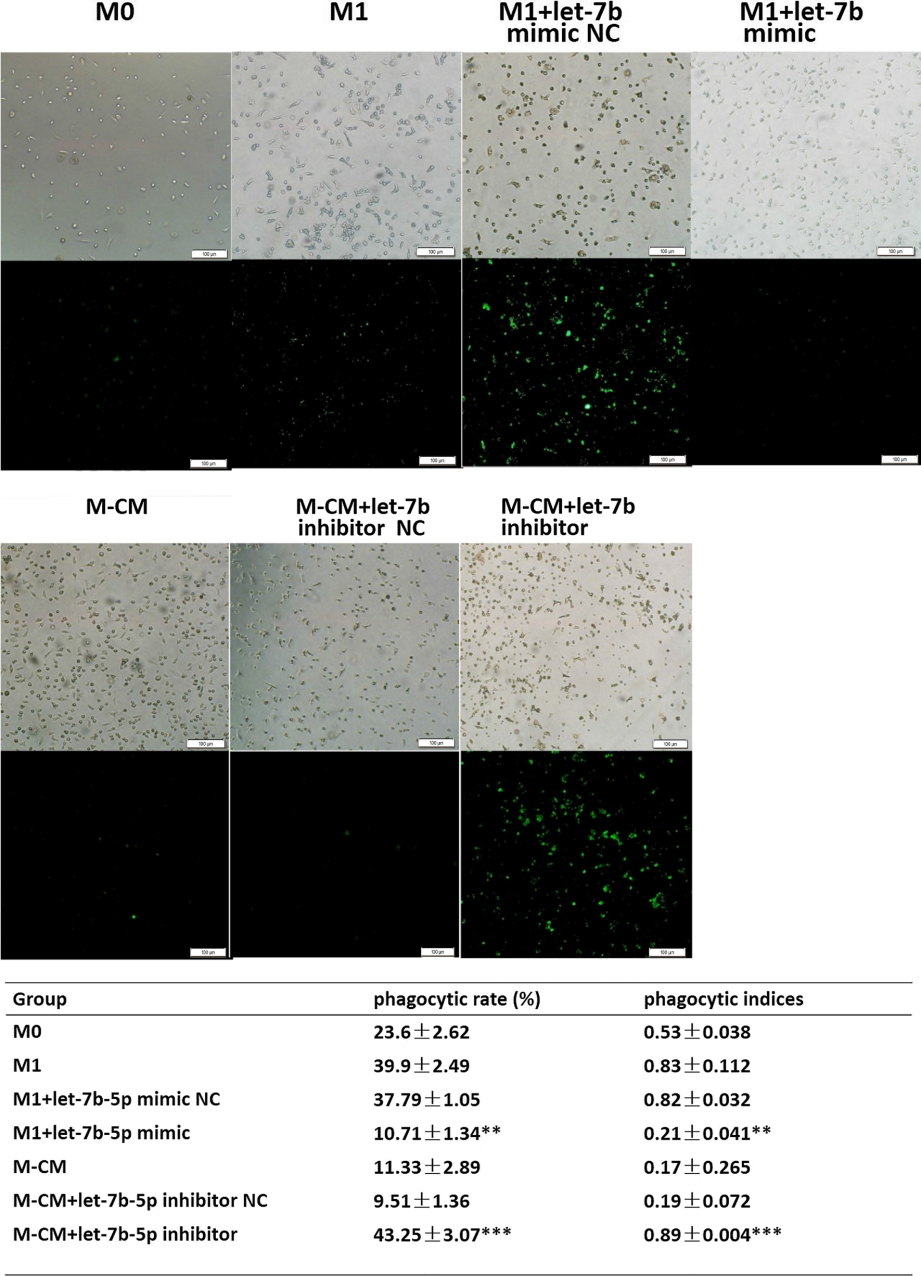
Let-7b-5p reduces phagocytosis by macrophages. After FITC-*Staphylococcus aureus* antigen was added to normal, cultured macrophages in each group at a ratio of 1:20 (*S. aureus*:macrophages), the mixture was shielded from light and incubated at 37 °C for 20 min. The supernatant was removed, and we recorded phagocytosis by macrophages in each group and quantified them by measuring the numbers of macrophages with FITC-*S. aureus* antigen in a field using the High Throughput Connotation of Imaging System (original magnification, 100×). *P < 0.05; **P < 0.01; ***P < 0.001

To address the link between let-7b-5p and macrophage phagocytosis, we used a phagocytosis test to investigate the phagocytosis of macrophage subtypes. Our results showed that the ability to phagocytize *Staphylococcus aureus* was significantly decreased in M-CMs expressing high levels of let-7b-5p and in M1 treated with let-7b-5p mimics (*P < 0.01), whereas their phagocytic ability was significantly increased in M-CMs treated with let-7b-5p inhibitors and M1 (see Fig. 5). These results suggested that let-7b-5p affects phagocytosis by macrophages.

### Reversal of M-CMs by let-7b-5p inhibitors suppresses the proliferation of PCa cells

**Fig. 6:**
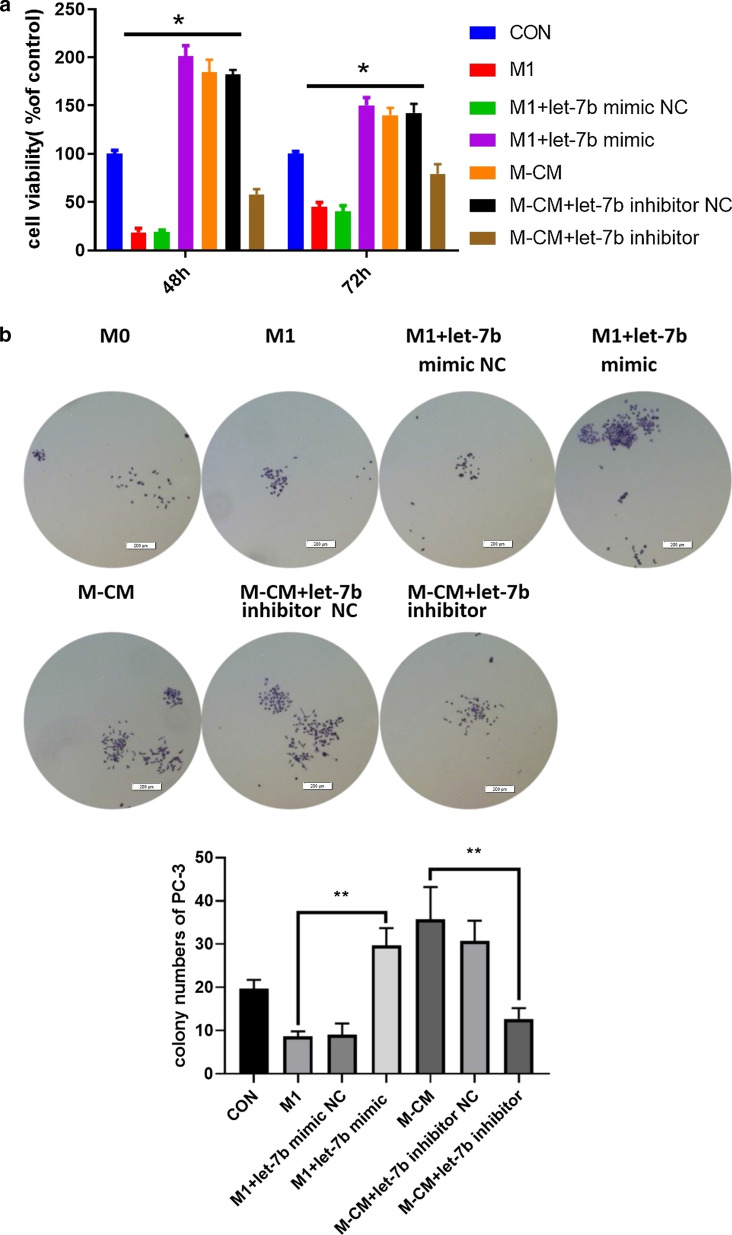
Reversal of M-CMs by downregulation of let-7b-5p inhibits the proliferation of PCa cells. M1 and M-CMs were transfected with let-7b-5p mimics or inhibitors, respectively, for 72 h. Then CM from macrophages was collected and added to the PC-3 cells. **a** PC-3 cells were exposed to CM from macrophages of different groups or RPMI 1640 medium as a control for 48 or 72 h. We measured viability of the PC-3 cells with the CCK-8 assay, and counted the number of viable cells relative to the control group. *P < 0.05; **P < 0.01. **b** After 2 weeks of incubation, CM from M-CMs treated with let-7b-5p inhibitors significantly decreased PC-3 colony number compared with the conditioned medium from M-CM. Each value represents the mean ± SEM. *P < 0.05, **P < 0.01

Macrophages express relatively large numbers of cytokines involved in the inhibition of proliferation of various cancer cells. Thus, we further determined whether let-7b-5p was involved in pro-tumor functions in macrophages by incubating human PCa cells with conditioned medium (CM) from macrophages treated with let-7b-5p inhibitors or mimics, or with negative control. As shown in Fig. 6a, CM from M1 plus let-7b-5p mimics exerted greater stimulatory effects on PC-3 cells relative to those from M1, and CM from M-CMs treated with let-7b-5p inhibitors led to the significant suppression of PC-3 cell proliferation compared to that from M-CMs. We further measured the effect of these CM on the colony-forming abilities of PC-3 cells. The results indicated that the colony-forming ability of PC-3 cells was also markedly attenuated with CM from M-CM under let-7b-5p inhibition (Fig. 6b). These results suggested that macrophage differentiation modulated by let-7b-5p plays a critical role in PCa proliferation.

## Discussion

Events surrounding the mutual shift in the M1/M2 ratio constitute an exciting area in the current investigation of the TME. Macrophages can infiltrate tumors, and their adjacent normal tissues become TAMs, which have a phenotype similar to that of M2 [18], including inhibiting inflammation and promoting tissue remodeling and repair. A growing number of studies have revealed that macrophages manifest high functional plasticity and heterogeneity, and that the phenotype and functional shift of macrophages in the TME often affect the occurrence and development of disease. Gollapudi [6] demonstrated that the number of TAMs was highest in PCa, followed by prostatic intraepithelial neoplasia and benign tumor tissue—suggesting the potential role of TAMs in the development of PCa [19]. For example, the number of macrophages that infiltrate PCa is reportedly associated with the Gleason score, and is also a useful predictive factor of PCa progression after hormonal therapy [20, 21]. Although the reversal of TAMs is critical in promoting anti-tumor immunity, mediation of the M1/M2 transition remains unclear. In this study, we demonstrated that let-7b-5p modulated macrophage differentiation by interacting with SOCS1, leading to activation of the STAT pathway and secretion of pro-tumor cytokines. Additionally, the reversal of M2 differentiation by let-7b-5p inhibitors enhanced macrophage phagocytosis and potentially exhibited anti-tumor efficacy. These data support the possibility of a miRNA-based M1/M2 transition in immunotherapy for PCa.

MiRNAs are powerful intracellular regulators involved in regulating the development, differentiation, and maturation of immune cells such as T cells, DCs, and macrophages [22]. Data from miRNA-sequencing and microarrays in M1, M2, and monocytes indicated that macrophage polarization is dynamically regulated by several miRNAs [8, 23]. As an important family of miRNAs, let-7b-5p participates in the growth, proliferation, invasion, and metastasis of cancer by regulating the expression of a variety of oncogenes and cytokines [24]. Specifically, let-7a, let-7b, and let-7c have been identified as potential tumor suppressors that directly target the mRNAs of genes involved in the cell cycle and signal transduction pathways, thus contributing to the initiation and development of PCa [25, 26]. Let-7c is also associated with macrophage differentiation [27], although the role of let-7b-5p in the shift of M1/M2 in PCa remains largely unelucidated.

Our previous study showed that let-7b was highly expressed in M-CMs, which manifest a M2-like phenotype [17]. To further explore the mechanism underlying let-7b-5p regulation of the M1/M2 transition, we induced M1/M-CM macrophages with monocytes from peripheral blood mononuclear cells (PBMCs) and transfected let-7b-5p mimics or let-7b-5p inhibitors into M1 and M-CMs, respectively, to observe the effects (Fig. 1a). Our results showed that CD206 expression in M-CMs (11.4%) treated with let-7b-5p inhibitors was almost at the same level as that in M1 (14.3%), whereas CD206 levels in M1 treated with let-7b-5p mimics (80.2%) were similar to those in M-CMs (78.3%) (Fig. 1b). Additionally, we observed that in M-CMs treated with let-7b-5p inhibitors, TNF-α, IL-10, and IL-13 were downregulated, and that IL-12 was upregulated, while in M1 treated with let-7b-5p mimics, the expression of TNF-α, IL-10, and IL-13 was increased, and the expression of IL-12 was decreased (Fig. 2). M1 macrophages are characterized by an IL-1R^high^ and IL-12^high^ phenotype, but M2 macrophages are characterized by an IL-10^high^ and IL-12^ l^°^w^ phenotype. This switch in macrophage subtype is now thought to be involved in the secretion of inflammatory cytokines and the expression of some specific markers for M2 macrophages such as CD206 and CD163. Due to the changes in CD206 and cytokines in the macrophages, we assumed that let-7b-5p modulated the M1/M2 transition by regulating the expression of inflammatory cytokines. Our data indicated that let-7b-5p was involved in the regulation of the M1/M2 phenotype transition in PCa.

The inflammatory-signaling pathway is considered to be the key pathway in macrophage polarization. Corresponding inflammatory cytokines are also reportedly involved in tumor occurrence and development [6]. Therefore, maintaining the homeostasis of these signaling molecules is crucial. As an important negative regulator of the signal-transduction pathway, the SOCS family principally regulates the expression of cytokines by inhibiting the JAK/STAT-signaling pathway [28].

The JAK/STAT-signaling pathway regulated by SOCS1 is a signal-transduction pathway stimulated by cytokines. It participates in many important biologic processes such as proliferation, differentiation, apoptosis, and immune regulation [29, 30]. Because SOCS1 is the candidate target gene for let-7b-5p according to the bioinformatics prediction algorithm TargetScan, we compared the levels of SOCSI protein in M-CMs and M1 after let-7b-5p intervention, and found that let-7b-5p was negatively correlated with SOCS1 expression. In M-CMs transfected with let-7b-5p inhibitors, the expression of SOCS1 was significantly upregulated as with M1, whereas in M1 transfected with let-7b-5p mimics, the expression of SOCS1 was similar to that for M-CMs (Fig. 3a). Using luciferase assays, we further confirmed that let-7b-5p directly targeted the SOCS1 3′-untranslated region (UTR). After co-transfection with let-7b-5p and the SOCS1-3′-UTR, the fluorescence intensity of the reporter gene significantly diminished, while the intensity remained unchanged in the MUT group of the SOCS1-3′-UTR (Fig. 3b, c).

As a particularly potent inhibitor of JAK1 and JAK2 [31], SOCS1 mediates the activities of STAT1 [32], STAT3 [33], and STAT5a [34]—which regulate the expression of a large number of cytokines that include IL-9, IL-10, IL-21, IL-22, and TNF-α [35]. For example, STAT3 in TAMs from breast cancer is overactivated, which inhibits the expression of IL-12 and significantly promotes the secretion of TNF-α [36]. To investigate the downstream targets of SOCS1, we next analyzed the phosphorylation status of STAT family proteins in macrophages after let-7b-5p intervention using the CST signal-pathway chip. Our results showed that let-7b-5p promoted the phosphorylation of STAT1, STAT3, and STAT5A proteins in macrophages (Fig. 4). In M1 transfected with let-7b-5p mimics, the expression of p-STAT1, p-STAT5a, and p-STAT3 was significantly increased (**P < 0.01)—similar to that in the M-CM group. Conversely, the expression of p-STAT1, p-STAT3, and p-STAT5a was significantly decreased in M-CMs transfected with let-7b-5p inhibitors (**P < 0.01)—similar to that in the M1 group. In RAW264.7 cells, galectin-9 showed similar results, regulating macrophage polarization through STAT1 and STAT3 [37]. These results indicated that let-7b-5p mediates the M1/M2 transition via the SOCS1/STAT-signaling pathway.

Phagocytosis, antigen processing, and presentation of macrophages are important components of immune surveillance. M2 macrophages or TAMs with M2-like properties display pro-tumor functions by virtue of their poor ability in the phagocytosis of dead cells, matrix, cell debris, and pathogens. To further investigate the effects of let-7b-5p on macrophage function, we analyzed the phagocytosis by macrophages after let-7b-5p intervention. As expected, both M-CMs transfected with let-7b-5p inhibitors and M1 showed higher phagocytic levels compared to the other groups, and M1 transfected with let-7b-5p mimics exhibited a lower phagocytic rate similar to that of M-CMs (Fig. 5).

Macrophage cytokines have the ability to enhance the proliferation of normal prostate epithelial cells [38]. Classically activated M1 macrophages—secreting a variety of pro-inflammatory factors such as IL-12 and IFN*-γ*—are considered to be anti-tumor factors. In contradistinction, TAMs promoted the proliferation, invasion, and metastasis of tumor cells. Herein we observed the role of these macrophages in the proliferation of PC-3 cells. Our data demonstrated that CM from M-CMs treated with let-7b-5p inhibitors significantly suppressed the proliferation of PC-3 cells compared with M-CMs (Fig. 6), suggesting that the inhibition of let-7b-5p can reverse M2 differentiation to some extent and inhibit PCa proliferation. One explanation for this phenomenon is that after M-CMs were treated with let-7b-5p inhibitors, IL-10, IL-13 and TNF-α were down-regulated, while IL-12 was significantly up-regulated. IL10 is an anti-inflammatory, immune-suppressing cytokine, and IL10 serum levels in cancer patients are positively correlated with Gleason scores [39]. In the early 2000s, Stearns et al. reported that IL-10 treatment of PCa cell lines increased TIMP1 and decreased MMP1 and MMP2 synthesis, and contributed to PCa progression [40]. IL-13 is also important in prostate tumorigenesis, as receptors for IL-13 are expressed by several cancers including prostate carcinoma. In 2015, Devikala et al. reported that the expression of IL-13 was positively correlated with human prostatic tumors, and that tumors obtained from *TRAMP*^+^*Il13*^*−/−*^ mice were smaller than those obtained from *TRAMP*^+^*Il13*^+/−^ or *TRAMP*^+^*Il13*^+*/*+^ mice [41]. A series of experiments in murine cancer models have suggested that TNF-α KO mice are resistant to chemically induced carcinogenesis of the skin [42], and that inhibition of stromal cell TNF-α production decreases the incidence of liver tumors [43]. In addition, IL-12 is a cytokine with both immunostimulatory and antiangiogenic effects, and accumulated evidence has clearly illustrated the importance of endogenous IL-12 in preventing cancer initiation, growth, and metastasis [44, 45]. Since these cytokines in the TME have the capacity to mediate the growth and metastasis of tumor cells, we speculate that these cytokines synergize to suppress proliferation of PCa.

## Conclusions

Collectively, these results confirm that let-7b-5p regulates the M1/M2 transition by regulating the activity of the SOCS1/STAT pathway, ultimately affecting PCa proliferation. Our study expands the role of let-7b-5p directly or indirectly in mediating anti-tumor immunity. As a novel regulator of the M1/M2 switch, let-7b-5p constitutes a promising target in the design of future therapies for macrophage-mediated pathologies.

## Materials and methods

### Cell culture

Human PCa cells (PC-3) were obtained from the American Type Culture Collection (Manassas, VA, USA) and cultured in RPMI-1640 medium (Life Technologies Corporation, Grand Island, NY, USA), supplemented with 10% fetal bovine serum (FBS) (Life Technologies, Burlington, ON, Canada) at 37 °C in 5% CO_2_. Human macrophages were induced by blood monocytes from healthy male donors; human monocytes were isolated from PBMCs using anti-CD14 magnetic beads (Miltenyi Biotec, Bergisch Gladbach, Germany) according to the manufacturer’s protocol. Purified monocytes (1 × 10^6^) were seeded in a 6-well plate and incubated for 7 days in RPMI 1640 supplemented with 10% FBS and 50 ng/mL M-CSF (Peprotech, Rocky Hill, NJ, USA) to obtain macrophages. Subsequently, macrophages were induced with 100 ng/mL of lipopolysaccharides (Peprotech) plus 100 ng/mL of IFN-γ (Peprotech) or 50% CM from PC-3 cells, respectively, for 48 h to obtain M1 and the prostatic M-CM subtype; M0 cells were obtained by treatment with serum-free medium for 48 h. After growing to the logarithmic phase, we cultured cells in serum-free medium for 24 h, collected the supernatant—which was centrifuged and filtered—and added 10% FBS to obtain CM.

### Transfection assay

The chemically modified hsa-let-7b-5p inhibitors, mimics, and corresponding NC oligonucleotides were purchased from RiboBio Corporation (Guangzhou, China). M1 macrophages were transfected with 50 nM let-7b-5p mimics, 50 nM NC, or remained untreated using FuGENE® 6 Transfection Reagent (Promega Corporation, Madison, WI, USA) according to the manufacturer’s instructions, while M-CMs were transfected with 100 nM let-7b-5p inhibitors or 100 nM NC. The cells were harvested after 72 h of transfection for subsequent experiments.

### Immunofluorescence staining

After the supernatants were removed, we aliquoted the macrophages into a 96-well plate and washed them with PBS three times, after which 200 µL of 4% paraformaldehyde was added to each well for 15 min at room temperature. After the cells were gently washed 3 times with PBS, we labeled the macrophages with primary antibody (CD206-PE as the M2 marker) (eBioscience, San Diego, CA, USA) for 10 min at room temperature, stained the cells, washed them twice with PBS, and added RPMI 1640 medium. The expression of CD206 was detected using a High Throughput Connotation of Imaging System (Molecular Devices, San Jose, CA, USA).

### ELISA

The whole-cell protein lysates from macrophages of each experimental group were isolated using RIPA, and the protein concentrations were determined using the Bradford protein assay (Bio-Rad, Hercules, CA, USA). We used ELISA kits (China Union Biology, Beijing, China) to analyze the expression of SOCS1 in each experimental group according to the manufacturer’s protocol. The concentration of SOCS1 was measured by comparing its optical density value with the corresponding standard curve.

### Quantitative RT-PCR

We extracted total RNA using the Invitrogen Trizol Reagent (Life Technologies Corporation, Carlsbad, CA, USA), and then used it as a template for cDNA synthesis. For miRNA, 100 ng of total RNA was reverse transcribed directly using MicroRNA^TM^ first-strand synthesis (Tokara, Kyoto, Japan) to synthesize cDNA. Quantitative PCR (qPCR) was performed in the Bio-RAD CFX96TM Real-Time System (Bio-Rad Laboratories, Inc., Hercules, CA, USA) using the SYBR Green PCR Master Mix (Tokara, Kyoto, Japan) according to the manufacturer’s protocol. All primers used in this study are shown in Additional file 1: Table S1.

### CST signal-pathway chip

Macrophage cells from each group were harvested and lysed, and the supernatant was collected. After the protein concentration was determined by the Bio-Rad protein assay, 40 µg of cell lysates from each group was used to analyze the expression of macrophage-signaling proteins via the CST signal-pathway chip (Cell Signaling Technology, Inc., Danvers, MA, USA) according to the manufacturer’s instructions, and the derived data were visualized using the Bio-Rad imager (Bio-Rad Laboratories).

### Phagocytosis test

*Staphylococcus aureus* was cultured and adjusted to 1 × 10^9^/mL, mixed with an appropriate amount of FITC powder, and then kept at 4 °C in a dark place for 4 h to obtain FITC-*S. aureus* antigen. After FITC-*S. aureus* antigen was added to the normal, cultured macrophages in each group at a ratio of 1:20 (*S. aureus*:macrophages), the mixture was shielded from light and incubated at 37 °C for 20 min. Afterward, the supernatant was removed, and the cells were washed three times. Finally, macrophage phagocytosis was detected and calculated for each group using the High Throughput Connotation of Imaging System. The percentage of phagocytosis (%) = the number of macrophages phagocytizing *S. aureus*/the total number of macrophages counted × 100%.

### Cell counting kit-8 assay

We detected cellular viability according to the instructions provided with the Cell Counting Kit-8 (CCK-8; Beyotime Institute of Biotechnology, Beijing, China). PC-3 cells (100 µL) were seeded at 5 × 10^4^/mL in 96-well plates and cultured to 80% confluency. The medium was replaced with CM from the macrophages of the different groups and then incubated for 48 or 72 h. Then 10 µL of CCK-8 reagent was added to each well, and cell growth was measured at 490 nm using the SpectraMax M4 Multimode Microplate reader (Molecular Devices, San Jose, CA, USA). The number of viable cells is presented relative to the control group.

### Dual-luciferase reporter assay

WT containing let-7b-5p binding sites or the MUT SOCS1-3′UTR mutated at the let-7b-5p binding sites was inserted into the pmiR-RB-Report™vector (Ribobio Corporation, Guangzhou, China) according to the manufacturer’s instructions. Then 50 nM of let-7b-5p mimics or mimic control (Guangzhou RiboBio Co., Ltd.) and WT or the MUT 3′-UTR of SOCS1 were co-transfected into 293 T cells using the Lipofectamine^®^ 2000 reagent (Invitrogen, Thermo Fisher Scientific, Inc.) according to the manufacturer’s protocol. After cells were cultured at 37 °C for 48 h, we analyzed luciferase activities using the Dual-Luciferase Reporter Assay system (GeneCopoeia, Inc., Rockville, MD, USA), and the activity of firefly luciferase was normalized to the corresponding *Renilla* luciferase activity. Each experiment was performed three times.

### Colony-formation assay

PC-3 cells were seeded at a cellular density of 100 cells per well in 24-well plates and cultured with CM from the macrophages of the different groups at 37 °C with 5% CO_2_ for 2 weeks. The cells were then fixed in methanol and stained with 0.1% crystal violet for 20 min. We counted colonies using ImagePro Plus 6.0 software (Media Cybernetics, Silver Spring, MD, USA).

### Data processing

We used Prism 5.0 software for statistical analysis. 1-way analysis, followed by Tukey's post hoc multiple comparison tests was applied to determine statistical significance. Significance values are indicated as *(P < 0.05), **(P < 0.01).

## Supplementary information

**Additional file 1: Table S1.** A list of all the primers.

## Data Availability

The data used to support the findings of this study are available from the corresponding author upon request.

## Abbreviations

TME: Tumor microenvironment
TAM: Tumor-associated macrophage
PCa: Prostate cancer
SOCS: Suppressor of cytokine signaling
JAK/STAT: Janus kinase/signal transducer and activator of transcription
ELISA: Enzyme-linked immunosorbent assay
M-CM: Macrophages induced by conditioned medium from PC-3 cells
