# Supplementary material for: Inhibition of let-7b-5p contributes to an anti-tumorigenic macrophage phenotype through the SOCS1/STAT pathway in prostate cancer
Source: Cancer Cell Int. 2020 Sep 29;20:470. doi: 10.1186/s12935-020-01563-7 (PMC7526222; doi:10.1186/s12935-020-01563-7)
Supplement: Supplementary file 1 — Additional file 1: Table S1. A list of all the primers. [file 12935_2020_1563_MOESM1_ESM.docx]

Table S1. A list of all the primers

| Primer name |  | Primer sequences |
| --- | --- | --- |
| GAPDH forward primer  GAPDH reverse primer  TNF-alpha forward primer  TNF-alpha reverse primer  IL-12 forward primer  IL-12 reverse primer  IL-10 forward primer  IL-10 reverse primer  IL-13 forward primer  IL-13 reverse primer  U6 forward primer  U6 reverse primer  let-7b forward primer  let-7b reverse primer | | 5′-GACCCCTTCATTGACCTCAAC-3′  5′-CTTCTCCATGGTGGTGAAGA-3′  5′-CTGGGCAGGTCTACTTTGGG-3  5′-CTGGAGGCCCCAGTTTGAAT-3  5'-CATTGAGGTCATGGTGGATG-3'  5'-CAAGTTCTTGGGTGGGTCAG-3'  5'-AGAACCAAGACCCAGACATCA-3'  5'-GCATTCTTCACCTGCTCCAC-3'  5'-ATCCTCTCCTGTTGGCACTG-3'  5'-CTGGTTCTGGGTGATGTTGAC-3  5′-CTCGCTTCGGCAGCACA-3′  5′-AACGCTTCACGAATTTGCGT-3′  5'- GCGCTGAGGTAGTAGGTTGTG -3'  5'-GTGCAGGGTCCGAGGT-3' |
